# Ischemic Colitis Revealing Polyarteritis Nodosa

**DOI:** 10.1155/2013/741047

**Published:** 2013-12-09

**Authors:** Amira Hamzaoui, Noureddine Litaiem, M. Smiti Khanfir, Sofiene Ayadi, Haifa Nfoussi, M. H. Houman

**Affiliations:** ^1^Department of Internal Medicine, La Rabta, 1007 Tunis, Tunisia; ^2^Department of Surgery A, La Rabta, 1007 Tunis, Tunisia; ^3^Department of Anatomopathology, La Rabta, 1007 Tunis, Tunisia

## Abstract

Ischemic colitis is one of the most common intestinal ischemic injuries. It results from impaired perfusion of blood to the bowel and is rarely caused by vasculitis. We report a case of ischemic colitis revealing polyarteritis nodosa (PAN) in a 55-year-old man. Histological examination of the resected colon led to the diagnosis of PAN.

## 1. Introduction

A 55-year-old man admitted with acute abdomen surgery proved to be necessary. Histological examination of the pathologic specimens revealed fibrinoid necrosis and destruction of the internal lamina in small and medium size arteries. Although the gastrointestinal tract is frequently involved, it is rare for PAN to be accompanied by severe ischemic colitis as initial presentation [1, 2].

## 2. Case Report

A 55-year-old man, with recurrent abdominal pain since 10 months treated with antispasmodic drugs, was admitted in Rabta's Department of Surgery (A), because of fever, abdominal pain, and bloody stool. Abdominal examination revealed mild rebound tenderness.

Laboratory tests revealed severe inflammation. Upper gastrointestinal endoscopy was normal. Colonoscopy showed “ulcerative and bleeding mucosa in sigmoid colon.”

CT-scan revealed “dilatation of sigmoid colon, wall thickening, and hyperattenuation. Colon was distended upstream.”

At laparotomy, the colon appeared cyanotic. Gentle palpation of sigmoid colon caused violaceous discoloration of the bowel. Pulsations were left in the celiac, superior mesenteric, and inferior mesenteric arteries. The entire colon was resected and ileostomy was created.

**Figure 1:**
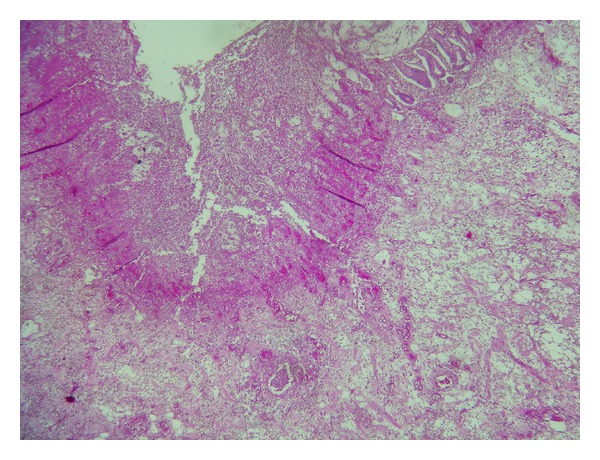
Mucosa with necrosis and hemorrhagic lesions. Submucosa dissociated with exudatif rearrangement (HE ×40).

**Figure 2:**
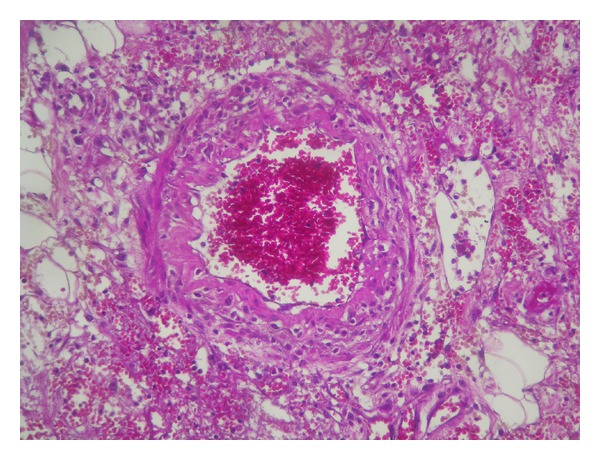
Leukocytoclastic vasculitis (HE ×400).

**Figure 3:**
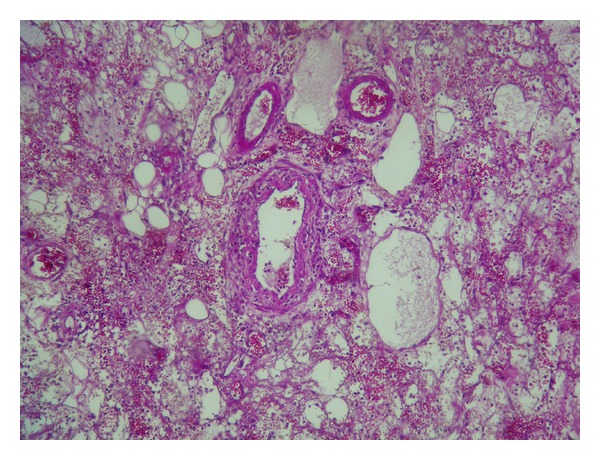
Fibrinoid necrosis and leucocytoclastic vasculitis (HE ×250).

**Figure 4:**
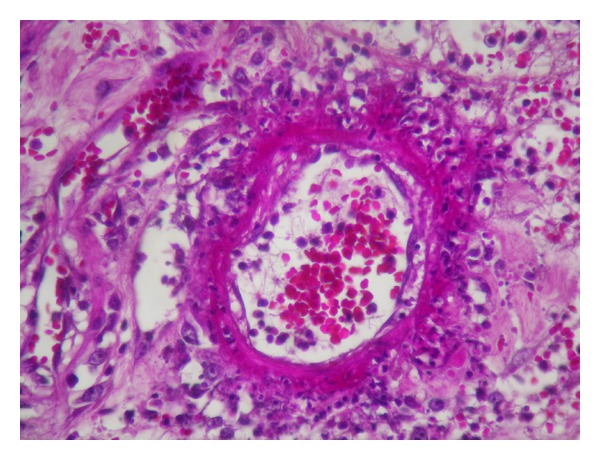
Fibrinoid necrosis with inflammatory infiltrate rich in neutrophils (HE ×400).

Histological examination of the pathologic specimens revealed “fibrinoid necrosis and destruction of the internal lamina in small and medium-size arteries which are rich in plasma cells, lymphocytes, and neutrophils” (Figures 1, 2, 3, and 4).

He was diagnosed with PAN and was admitted to our department.

Physical examination revealed reduction in tactile sensitivity in the territory of the right peroneal nerve and both ulnar nerves; the electromyography showed multiple mononeuropathy.

Urinalysis, coagulation profile, serum electrolytes and enzymes, serology of hepatitis B and C, antineutrophil cytoplasmic antibody (ANCA), and serum tests for rheumatic diseases were normal.

Computed tomography did not reveal microaneurysms.

Treatment included 3 intravenous pulses of methylprednisolone, 60 mg/day of prednisone, and 12 monthly intravenous pulses of cyclophosphamide. After discharge, a progressive withdrawal of prednisone was accomplished. One year after, PAN has not relapsed.

## 3. Discussion

We have presented a case of PAN revealed by ischemic colitis necessitating acute surgical intervention.

Polyarteritis nodosa (PAN), first described in 1866 by Kussmaul and Maier [1], is systemic necrotizing vasculitis that predominantly affects medium sized arteries and is primary in most patients but is the consequence of viral infections, mainly hepatitis B virus (HBV), in some. Biopsy material can prove the diagnosis, especially if an affected area or lesion of the skin, muscle, or other tissue is available. The characteristic histopathological changes of PAN are fibrinoid necrosis of the walls of medium or small arteries, with a marked inflammatory response within or surrounding the vessel [2].

PAN involves the gastrointestinal (GI) tract in more than 50% of patients at some time during its course [3, 4].

Clinically apparent ischemic disease of the small bowel is the frequent site of involvement. The colon is less commonly involved [4].

The presentation of colonic PAN may mimic inflammatory bowel disease in young patients and atherosclerotic ischemic colitis in older ones [5]. Thus, any GI symptoms which are preceded by fever, weight loss, myalgia, or arthralgias should raise the possibility of vasculitis. In those patients who do not require an urgent intervention, a deep endoscopic biopsy must be taken. This can give the diagnosis without the need for surgery.

When studying 342 patients with PAN, Pagnoux found that GI involvement was present in 132 cases (37.9%) and was significantly more frequent in HBV related PAN (*P *< 0.001) [3]. GI manifestations requiring surgery were noted in 48 patients (13.8%). In multivariate analysis, Gl manifestations requiring surgery at diagnosis were associated with increased risk of death. Bourgarit et al. conclude that GI symptoms were most frequently associated with early death from HBV-PAN [6].

Although GI involvement is frequent in PAN, the isolated abdominal initial presentation is uncommon, and since 1975, only 9 cases have been published in the English language literature. They are summarized in Table 1 [7–15].

In the presence of acute abdominal signs, early surgery is warranted. In their absence, medical treatment should suffice. In all cases, close observation by a surgical team must be undertaken.

For many years, PAN treatment has involved administration of high-dose steroid with an additional cytotoxic agent, such as cyclophosphamide, to induce remission. In most patients, it is appropriate to treat aggressively. Once remission is achieved, maintenance therapy with daily or alternate-day low-dose prednisolone and oral azathioprine is frequently used for up to 18 months [16].

PAN, unlike some other vasculitides such as Wegener granulomatosis, appears to be a condition in which permanent remission can be achieved. Relapses can occur, but despite these, a real possibility of cure can be anticipated. However, if treatment is delayed or inadequate, life-threatening complications can occur due to the vasculitic process.

## 4. Conclusion

Although the gastrointestinal tract is frequently involved with PAN, it is extremely rare for PAN to be accompanied by ischemic colitis, particularly at the initial presentation.

## Figures and Tables

**Table 1:** Summary of reported cases of PAN presenting with ischemic colitis.

*N* [Reference]	Year	Age/sex	GI symptoms	Extra GI symptoms	Finding on RS	Perforation	Surgery	Outcome
1 [7]	1979	76/M	Bloody diarrhoea	Myalgia; weight loss; fever	Colitis	No	Yes	D
2 [8]	1982	52/M	None	Myalgia; weight loss	—	No	Yes	S
3 [9]	1984	58/M	Diarrhoea; abdominal pain	Fever	Colitis	Yes	Yes	S
4 [10]	1991	28/M	Diarrhoea; abdominal pain	None	Colitis	No	Yes	S
5 [11]	1994	48/M	Abdominal pain; occult blood +	Myalgia; fever; weight loss	Colitis	Yes	Yes	S
6 [12]	1994	78/F	Abdominal pain and bloody stool	None	—	No	Yes	—
7 [13]	1996	38/F	Bloody diarrhoea; abdominal pain	Myalgia; fever; weight loss	Colitis	No	No	S
8 [14]	1999	60/M	Abdominal pain Bloody diarrhoea	None	Colitis	Yes	Yes	—
9 [15]	2008	70/M	Abdominal pain Bloody stool	None	Colitis	Yes	Yes	S
Our case	2010	55/M	Abdominal pain and bloody stool	Fever neuropathy	Colitis	No	Yes	S

M: male; F: female; GI: gastrointestinal, RS: rectosigmoidoscopy; S: survived; D: died.

## References

[1] Kussmaul A, Maier R Uber eine bisher nicht beschreibeneeigenthumliche ArtermenerKrankung (periarteritis nodosa), die mit Morbus Brightii und rapid fortsch retender allgemeiner Muskellahmung einhergeht. *Deutsches Archiv für Klinische Medizin*.

[2] Churg A, Ball GV, Bridges SL (2008). Pathologic features of vasculitis. *Vasculitis. 2*.

[3] Pagnoux C, Seror R, Henegar C (2010). Clinical features and outcomes in 348 patients with polyarteritis nodosa: a systematic retrospective study of patients diagnosed between 1963 and 2005 and entered into the French Vasculitis Study Group database. *Arthritis and Rheumatism*.

[4] Ebert EC, Hagspiel KD, Nagar M, Schlesinger N (2008). Gastrointestinal involvement in polyarteritis nodosa. *Clinical Gastroenterology and Hepatology*.

[5] Tanakaya K, Konaga E, Takeuchi H (2001). Penetrating colon ulcer of polyarteritis nodosa: report of a case. *Diseases of the Colon and Rectum*.

[6] Bourgarit A, Le Toumelin P, Pagnoux C (2005). Deaths occurring during the first year after treatment onset for polyarteritis nodosa, microscopic polyangiitis, and Churg-Strauss syndrome: a retrospective analysis of causes and factors predictive of mortality based on 595 patients. *Medicine*.

[7] Wood MK, Read DR, Kraft AR, Barreta TM (1979). A rare cause of ischemic colitis: polyarteritis nodosa. *Diseases of the Colon and Rectum*.

[8] Meyer GW, Lichtenstein J (1982). Isolated polyarteritis nodosa affecting the cecum. *Digestive Diseases and Sciences*.

[9] Lee EL, Smith HJ, Miller GL (1984). Ischemic pseudomembranous colitis with perforation due to polyarteritis nodosa. *The American Journal of Gastroenterology*.

[10] Gullichsen R, Ovaska J, Ekfors T (1991). Polyarteritis nodosa of the descending colon. *European Journal of Surgery*.

[11] Fort JG, Griffin R, Tahmoush A, Abruzzo AJL (1994). Muscle involvement in polyarteritis nodosa: report of a patient presenting clinically as polymyositis and review of the literature. *Journal of Rheumatology*.

[12] Ugai H, Nakata R, Takada H (1994). A case of ischemic colitis due to polyarteritis nodosa. *Nihon Ronen Igakkai Zasshi*.

[13] Ruiz-Irastorza G, Egurbide MV, Aguirre C (1996). Polyarteritis nodosa presenting as ischaemic colitis. *British Journal of Rheumatology*.

[14] Okada M, Konishi F, Sakuma K, Kanazawa K, Koiwai H, Kaizaki Y (1999). Perforation of the sigmoid colon with ischemic change due to polyarteritis nodosa. *Journal of Gastroenterology*.

[15] Gambino G, Rizzuto MR, Spallitta IS (2008). Isolated polyarteritis nodosa of the large bowel: a case report. *Chirurgia Italiana*.

[16] De Menthon M, Mahr A (2011). Treating polyarteritis nodosa: current state of the art. *Clinical and Experimental Rheumatology*.

